# Priming With Rhinovirus Protects Mice Against a Lethal Pulmonary Coronavirus Infection

**DOI:** 10.3389/fimmu.2022.886611

**Published:** 2022-05-30

**Authors:** Garrison Cox, Andres J. Gonzalez, Emmanuel C. Ijezie, Andres Rodriguez, Craig R. Miller, James T. Van Leuven, Tanya A. Miura

**Affiliations:** ^1^ Department of Biological Sciences, University of Idaho, Moscow, ID, United States; ^2^ Institute for Modeling Collaboration and Innovation, University of Idaho, Moscow, ID, United States

**Keywords:** rhinovirus, mouse hepatitis virus, interferon, respiratory virus co-infection, coronaviral infection, mouse model, coronavirus

## Abstract

Rhinoviruses (RV) have been shown to inhibit subsequent infection by heterologous respiratory viruses, including influenza viruses and severe acute respiratory syndrome-coronavirus 2 (SARS-CoV-2). To better understand the mechanisms whereby RV protects against pulmonary coronavirus infection, we used a native murine virus, mouse hepatitis virus strain 1 (MHV-1), that causes severe disease in the lungs of infected mice. We found that priming of the respiratory tract with RV completely prevented mortality and reduced morbidity of a lethal MHV-1 infection. Replication of MHV-1 was reduced in RV-primed mouse lungs although expression of antiviral type I interferon, IFN-β, was more robust in mice infected with MHV-1 alone. We further showed that signaling through the type I interferon receptor was required for survival of mice given a non-lethal dose of MHV-1. RV-primed mice had reduced pulmonary inflammation and hemorrhage and influx of leukocytes, especially neutrophils, in the airways upon MHV-1 infection. Although MHV-1 replication was reduced in RV-primed mice, RV did not inhibit MHV-1 replication in coinfected lung epithelial cells *in vitro*. In summary, RV-mediated priming in the respiratory tract reduces viral replication, inflammation, and tissue damage, and prevents mortality of a pulmonary coronavirus infection in mice. These results contribute to our understanding of how distinct respiratory viruses interact with the host to affect disease pathogenesis, which is a critical step in understanding how respiratory viral coinfections impact human health.

## Introduction

During the first year of the COVID-19 pandemic, public health measures that were implemented to halt severe acute respiratory syndrome-coronavirus 2 (SARS-CoV-2) transmission effectively reduced transmission of other respiratory viruses, particularly influenza viruses and respiratory syncytial virus (RSV) (1). Once societies began easing restrictions, the circulation of these viruses returned. In the case of RSV, this occurred out of its normal seasonal pattern (2, 3). As SARS-CoV-2 continues to circulate in human populations worldwide, there are overlapping incidences of COVID-19 and other respiratory viral infections (4). Circulation of rhinovirus (RV) was less affected than influenza viruses and RSV by public health measures intended to slow SARS-CoV-2 transmission, and RV has been frequently detected as a co-pathogen in COVID-19 patients (4, 5). Multiple studies have proposed that RV infections mediate viral interference with heterologous respiratory viruses, including influenza viruses and SARS-CoV-2 (6–8). While these studies are insightful, animal models in which disease outcomes can be studied in controlled systems of known viral doses, strains, and timing of infections are critical for determining viral interference mechanisms within a shared host.

Mouse hepatitis virus (MHV), in the Betacoronavirus genus, naturally infects the enteric tract of mice but various strains differ in tissue tropism and virulence. MHV strain 1 (MHV-1) has tropism for and causes disease in the respiratory tract (9–11). Mouse strain- and dose-dependent severity of MHV-1 infection allows researchers to study a broad range of pulmonary coronavirus disease severities under biosafety level 2 conditions. MHV-1 is particularly virulent in A/J and C3H/HeJ mice, causing severe lung pathology that resembles lethal infections by SARS-CoV or SARS-CoV-2 in addition to pathology in the liver, brain, heart, and kidneys (9, 12, 13). MHV-1 infection of BALB/c mice results in milder pulmonary disease with dose-dependent severity (9, 11, 14). Others have reported moderate disease upon infection of BALB/c mice with MHV-1 (9, 11). We have observed significant weight loss and 20% mortality of BALB/c mice infected with 2x10^3^ PFU of MHV-1 (14). The aim of these studies was to establish MHV-1 as a model for lethal pulmonary coronavirus infection in BALB/c mice and use it to study RV-mediated interference of coronavirus infection. We previously showed that inoculation of BALB/c mice with RV two days prior to inoculation with a lethal dose of influenza A virus (PR8) or pneumonia virus of mice (PVM) resulted in complete protection against mortality (14, 15). Here, we show that priming with RV reduced morbidity and prevented mortality of a lethal MHV-1 infection. This model system will be important for understanding the immunological mechanisms that underpin viral interference of pulmonary coronaviruses within hosts.

## Materials and Methods

### Ethics Statement

All procedures involving mice were approved by the University of Idaho’s Institutional Animal Care and Use Committee (Protocols 2017-29 and 2020-20), in compliance with the National Research Council Guide for the Care and Use of Laboratory Animals (16). Mice were monitored daily and were euthanized by an overdose of sodium pentobarbital if they reached humane endpoints, including loss of more than 20-25% of their starting weight or exhibiting severe clinical signs of disease.

### Cell Lines and Viruses

Murine fibroblast line 17Cl.1 (provided by Dr. Kathryn Holmes, University of Colorado Denver School of Medicine) and HeLa Cells (ATCC CCL-2) were grown in Dulbecco’s modified Eagle medium (DMEM) supplemented with 10% fetal bovine serum (FBS; Atlanta Biologicals), and antibiotic-antimycotic (ThermoFisher Scientific). The LA4 cell line derived from murine lung epithelial cells (ATCC CL-196) was grown in Ham’s F12 (Kaign’s modified) medium (F12K; Caisson) supplemented with 10% FBS and antibiotics. Mouse hepatitis virus strain 1 (ATCC VR-261) was grown in 17Cl.1 cells and titrated by plaque assay or TCID_50_ assay using 17Cl.1 cells. Human rhinovirus 1B (ATCC VR-1645) was grown in HeLa cells, concentrated by ultracentrifugation through 30% sucrose, and titrated by TCID_50_ assay using HeLa cells.

### Viral Infection

Six- to eight-week-old female BALB/c mice were purchased from Envigo and were allowed to acclimatize to the facility for 10 days prior to experimentation under animal biosafety level 2 conditions. Mice housed in individually vented cages with controlled light/dark cycles and regulated temperature were maintained by the University of Idaho Lab Animal Research Facilities and received food and water *ad libitum*. Mice were anesthetized with inhaled isoflurane and inoculated intranasally with 50 uL of virus or saline control (mock). Seven mice per group were inoculated with 7.6x10^6^ TCID_50_ units of RV or mock on day -2 and a lethal dose of MHV-1 (2x10^5^ PFU) on day 0. The mice were weighed daily and given a clinical disease score of 0 to 3 in each of four categories: ruffled fur, lethargy, labored breathing, and hunched posture. The daily scores were totaled for each individual mouse and averaged across the group of mice.

Five mice per group (mock/MHV and RV/MHV) were euthanized on days 2 and 5 after MHV-1 inoculation to analyze viral loads, interferon gene expression, histopathology in the lungs, and cellular infiltration of the airways. For titration of MHV-1, lung tissues were weighed and homogenized in 1 mL cold DMEM with 10% FBS and quantified by TCID_50_ assay using 17Cl.1 cells. Preliminary assays were done to demonstrate that RV does not interfere with titration of MHV-1 in 17Cl.1 cells (data not shown). RT-qPCR was performed on RNA isolated from whole lung tissues as described previously and below (14). Mouse lungs were lavaged twice with 1 mL cold phosphate buffered saline. Cells were counted on a hemocytometer with or without prior RBC lysis and spun on glass slides using a Shandon Cytospin. Slides were stained with HEMA3 staining kit for differential cell analysis. Lung tissues were fixed in formalin and processed and stained with hematoxylin and eosin as previously described (14). Images were acquired on a Zeiss axiolab microscope with Axiocam 105 color camera.

To inhibit type I interferon (IFN) signaling, mice were given 0.05 mg anti-IFNAR1 antibody (clone MAR1-5A3; Bio X Cell) intranasally with a sublethal dose of MHV-1(1x10^3^ PFU) and two days after inoculation. Control mice were given antibody of the same isotype (mouse IgG1*κ*, clone MOPC-21; Bio X Cell).

### Quantitative PCR

Lung tissues were stored in RNALater and RNA was extracted using TRIzol (Invitrogen) according to the manufacturer’s protocol. RNA was reverse transcribed using SuperScript IV VILO (Invitrogen). Quantitative PCR was performed using PowerUp SYBR green and StepOne Plus instrument (Applied Biosystems) using previously published primer pairs for IFN-β (17), Mx1 (18), and GAPDH (19). Fold change compared to values for mock-inoculated mice was calculated using the 2^-ΔΔCt^ method (20).

### Analysis of Viral Replication in LA4 Cells

LA4 cells were inoculated with RV and MHV-1 concurrently (RV+MHV) or sequentially with RV 6 h prior to inoculation with MHV-1 (RV/MHV), both viruses at a multiplicity of infection (MOI) of 1. Supernatant medium was collected every 6 h through the 24 h time point and at 48 h. Cells were removed by centrifugation, and MHV-1 titers in the medium were quantified by TCID_50_ assay using 17Cl.1 cells. LA4 cells seeded onto coverslips were inoculated with RV and MHV-1 concurrently and fixed with formaldehyde 18 h post-infection. Viral antigens were fluorescently labeled in triton X-100-permeabilized cells using monoclonal antibody against MHV nucleocapsid protein (provided by Dr. Julian Leibowitz, Texas A&M University) and donkey anti-mouse-555 (Jackson Immuno Research), and RV1B antiserum (ATCC, V-113-501-558) and donkey anti-guinea pig-488 (Jackson Immuno Research). Nuclei were stained with DAPI and images were obtained using a Nikon Eclipse E800 epifluorescence microscope and Nikon DS-Ri1 camera.

### Transcriptome Analysis

In all cases, LA4 cells were inoculated with viruses at an MOI of 1. For concurrent coinfection, cells were simultaneously inoculated with MHV-1 and RV and incubated for 12 h (MHV12+RV12). For sequential coinfections, cells were inoculated with RV for 12 h, followed by MHV-1 for an additional 12 h (RV24/MHV12) or vice versa (MHV24/RV12). Cells inoculated with RV or MHV-1 for 12 or 24 h as single virus controls and mock-inoculated cells served as negative controls. RNA isolation and microarray processing and analysis was done as previously described (21). Raw and processed data are available from NCBI Gene Expression Omnibus, accession numbers GSE89190 and GSE201471.

Differential gene expression between treatments was analyzed using normalized expression data and linear mixed-effect models followed by linear contrasts corrected for multiple comparisons (16). Expression was modeled as a function of infection, while probes for a particular gene were treated as random effects. The models used the nlme::lme function in R. The data contained three coinfection combinations in addition to the single virus infections, which were reported previously (16). The following nine *post-hoc*, two-sided contrasts were performed on the fitted models using the multcomp::glht function in R: each virus coinfection combination (MHV12+RV12, RV24/MHV12, MHV24/RV12) vs. mock and each pairwise combination of coinfection vs. single virus infection (MHV12+RV12 vs. MHV12, MHV12+RV12 vs. RV12, RV24/MHV12 vs. RV24, RV24/MHV12 vs. MHV12, MHV24/RV12 vs. MHV24, MHV24/RV12 vs. RV12). These tests had their p-values adjusted by the multcomp::summary.glht function according to their joint distribution.

### Statistics

Statistical analyses were performed using Prism9 software (GraphPad). Survival curves were compared using log rank Mantel-Cox curve comparison. Weight loss and clinical score data were analyzed using multiple Student’s *t* test with Holm-Sidak multiple-comparison correction. Viral titers and qPCR data were compared between groups using Student’s *t* tests without correction for multiple comparisons. Statistical analysis of transcriptome data is described above.

## Results

### Inoculation With RV Reduces Morbidity and Prevents Mortality of a Lethal MHV-1 Infection

**Figure 1:**
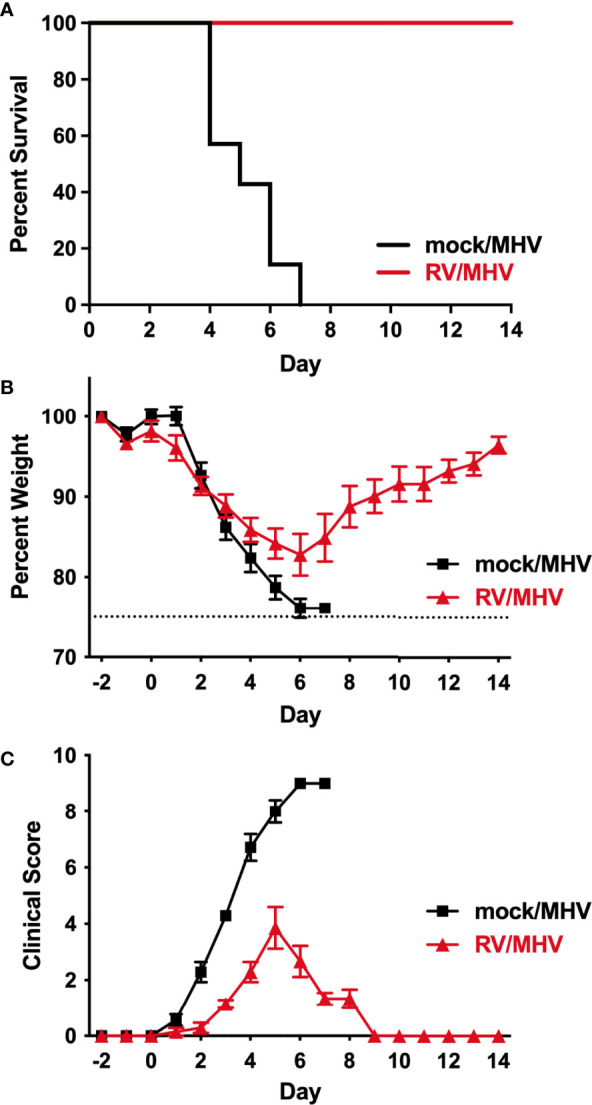
Priming with RV reduces morbidity and prevents mortality upon MHV-1 infection. Mice (n=7 per group) were inoculated intranasally with RV (7.6x10^6^ TCID_50_) or saline (mock) on day -2, and MHV-1 (2x10^5^ PFU) on day 0. Mice were monitored daily for **(A)** mortality (*p*=0.0004) **(B)** weight loss (*p*>0.05) and **(C)** clinical scores (days 2-7: *p*<0.001).

Based on our previous finding that BALB/c mice infected with 2x10^3^ PFU of MHV-1 experienced 20% mortality (14), we inoculated mice with 2x10^5^ PFU of MHV-1. This dose of MHV-1 resulted in 100% lethality (**Figure 1A**). In comparison to mice that received a mock inoculation two days before MHV-1 (mock/MHV), those that received RV (RV/MHV) were completely protected from mortality (**Figure  1A**). RV/MHV infected mice also had less severe morbidity, as determined by weight loss and clinical scores, compared to mock/MHV infected mice (**Figures 1B, C**). Although RV/MHV infected mice experienced significant weight loss, the rate of loss was lower than mock/MHV infected mice and they began regaining their body weight by day 7 after MHV-1 infection. Clinical signs of disease were delayed by two days and were much less severe in RV/MHV compared to mock/MHV infected mice. Clinical signs in mock/MHV infected mice included severely ruffled fur and hunched posture with mild to moderate lethargy and labored or shallow breathing. In contrast, clinical signs in RV/MHV infected mice were limited to mildly ruffled fur and hunched posture with occasional shallow breathing. Mock/MHV infected mice were humanely euthanized or succumbed to infection on days 4-7, while all RV/MHV infected mice survived through the end of the study (day 14).

### Inoculation With RV Reduces Replication of MHV-1 in the Lungs and Infiltration of Immune Cells into the Airways of Mice

**Figure 2:**
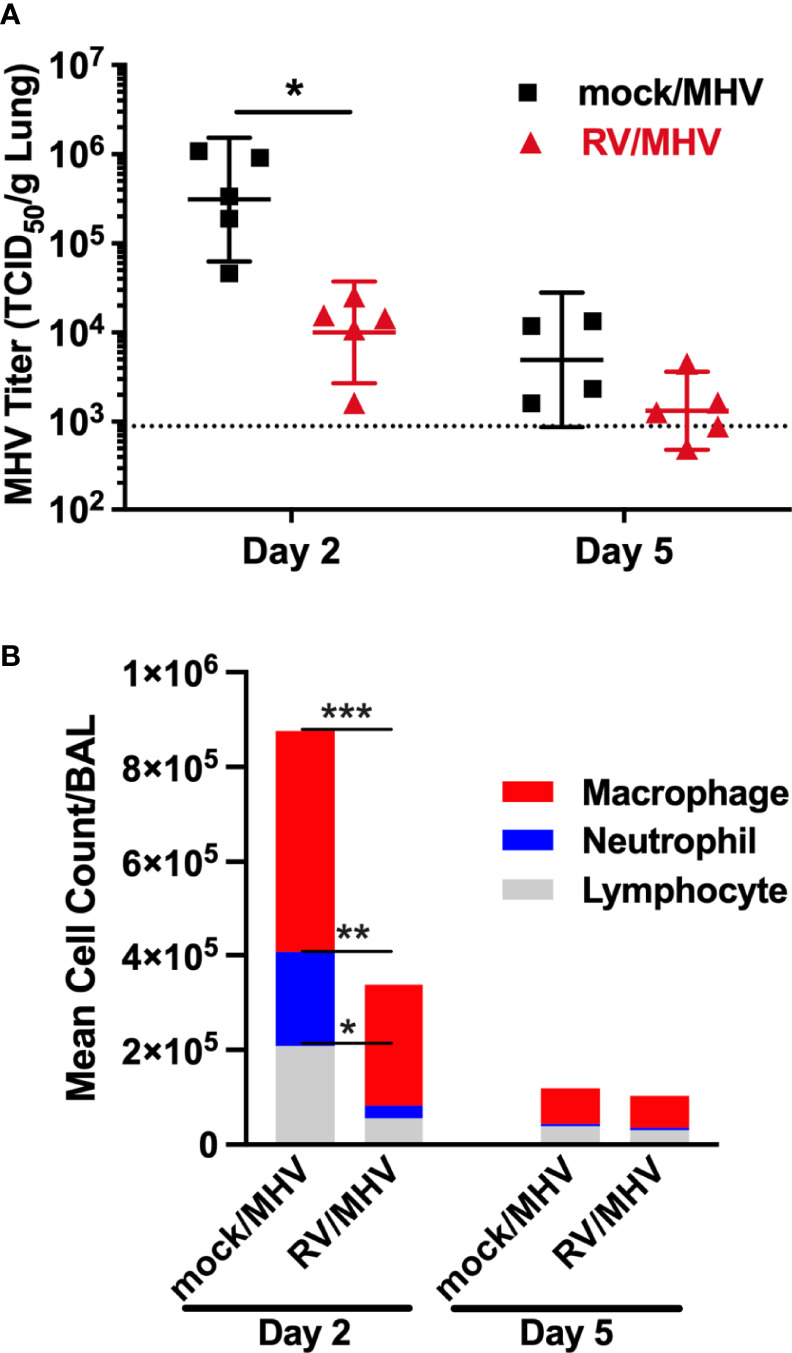
Priming with RV limits MHV-1 replication in the lungs and recruitment of leukocytes to airways. Mice (n=5 per group and time point) were inoculated intranasally with RV (7.6x10^6^ TCID_50_) or saline (mock) on day -2, and MHV-1 (2x10^5^ PFU) on day 0. **(A)** Lungs were collected, homogenized, and viral titers were determined by TCID_50_ assay using17Cl.1 cells. **(B)** Cells were counted and collected from bronchoalveolar lavage (BAL) fluid and stained with Wright-Giemsa stain for differential analysis. *p<0.05, **p<0.01, ***p<0.001.

To determine whether priming with RV inhibited replication of MHV-1, lungs from mock/MHV and RV/MHV infected mice were collected on days 2 and 5 after MHV-1 inoculation and MHV-1 titers were determined by TCID_50_ assay (**Figure 2A**). Although priming with RV did not completely prevent infection by MHV-1, titers on day 2 were approximately 1 log_10_ per gram of tissue lower in RV/MHV compared to mock/MHV infected mice. Mock/MHV infected mice that survived to day 5 had reduced viral loads in the lungs compared to day 2, indicating that lethality was not dependent on sustained viral replication. These data suggest that RV stimulates innate defenses that limit MHV-1 replication, resulting in reduced disease severity.

Bronchoalveolar lavage (BAL) fluid was collected from mice and the cellular content was stained for differential quantification. Mock/MHV infected mice had high numbers of cells in the airways on day 2 that consisted of ~60% macrophages, and ~20% each neutrophils and lymphocytes (**Figure 2B**). RV/MHV infected mice had lower overall cell numbers and the cells were predominantly macrophages with a reduced proportion of neutrophils compared to mock/MHV infected mice. Both groups had reduced total cell counts in the airways on day 5 after inoculation compared to day 2.

### Expression of IFN-β Corresponds With Higher MHV-1 Replication

**Figure 3:**
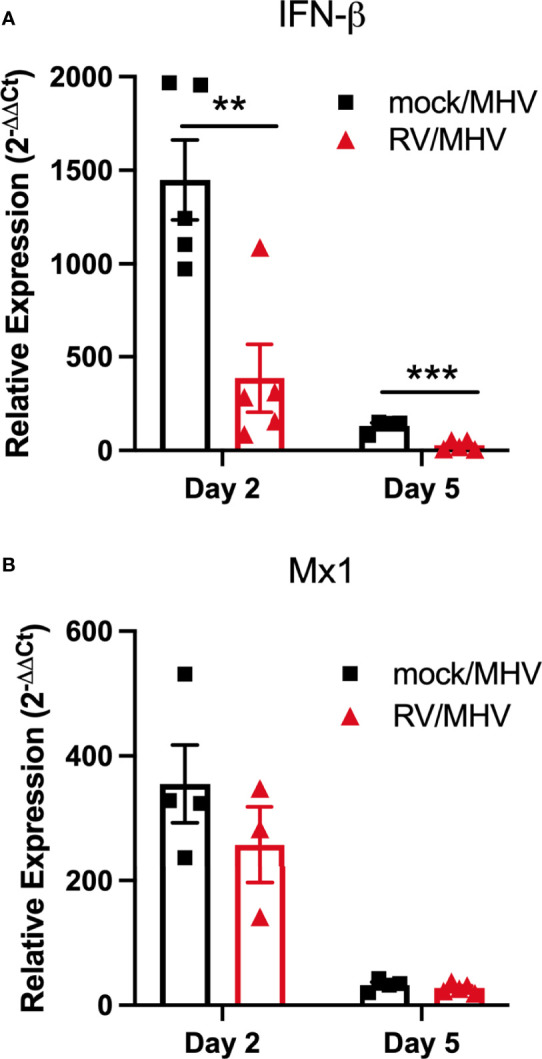
Priming with RV results in reduced expression of type I IFN response genes. Mice (n=5 per group and time point) were inoculated intranasally with RV (7.6x10^6^ TCID_50_) or saline (mock) on day -2, and MHV-1 (2x10^5^ PFU) on day 0. Total lung RNA was isolated and expression of **(A)** IFN-β and **(B)** Mx1 were quantified by RT-qPCR. **p<0.01, ***p<0.001.

Type I interferons, such as IFN-β, are antiviral cytokines that limit viral replication. Thus, we quantified levels of IFN-β and IFN-stimulated gene Mx1 mRNA from mock/MHV and RV/MHV infected lungs (**Figure 3**). Expression of IFN-β did not correspond with reduced viral titers in RV/MHV infected mice rather was significantly higher in mock/MHV infected mice (**Figure 3A**). Similarly, we previously observed limited induction of IFN-β expression upon inoculation with RV, compared to robust induction of an IFN response upon MHV-1 infection in BALB/c mice (14). The pattern of Mx1 expression was similarly high in mock/MHV infected mice, though not significantly higher than in RV/MHV infected mice (**Figure 3B**). These data suggest that IFN-β expression is strongly induced by MHV-1 infection but is not limiting viral replication early during infection and is not sufficient to protect from lethality in mock/MHV infected mice.

### Type I IFN Signaling Is Required for Survival Upon Infection by a Non-Lethal Dose of MHV-1

**Figure 4:**
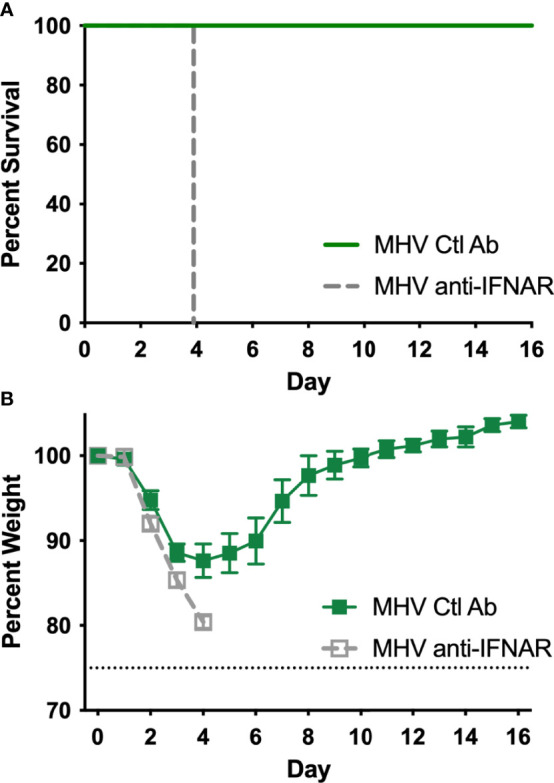
Signaling through the type I IFN receptor, IFNAR, is required for survival of sublethal MHV-1 infection. Mice (n=5 per group) were inoculated intranasally with MHV-1 (1x10^3^ PFU) and 0.05 mg of anti-IFNAR1 or control (Ctl) antibody on day 0. A second dose of antibody was given on day 2. **(A)** Mortality (*p*=0.0027) and **(B)** weight loss were monitored daily.

To determine if signaling through the IFNα/β receptor (IFNAR) is protective during a non-lethal infection with MHV-1, we used an anti-IFNAR1 blocking antibody to prevent IFNα/β-mediated responses. Mice were inoculated with a non-lethal dose of MHV-1 intranasally along with an irrelevant isotype control or anti-IFNAR1 antibody. Mice were given a second dose of antibody intranasally on day 2 post-infection. All mice that were given anti-IFNAR1 rapidly succumbed to MHV-1 infection by day 4, while the mice treated with control antibody all survived the MHV-1 infection (**Figure 4A**). Control antibody-treated mice lost weight early during MHV-1 infection and starting regaining weight after day 4 (**Figure 4B**). Although the robust production of IFN-β was not sufficient to protect against lethal MHV-1 infection (**Figure 3**), type I IFN signaling was required for protection against a non-lethal dose (**Figure 4**).

### Priming by RV Limits Pulmonary Inflammation and Hemorrhage Upon MHV-1 Infection

**Figure 5:**
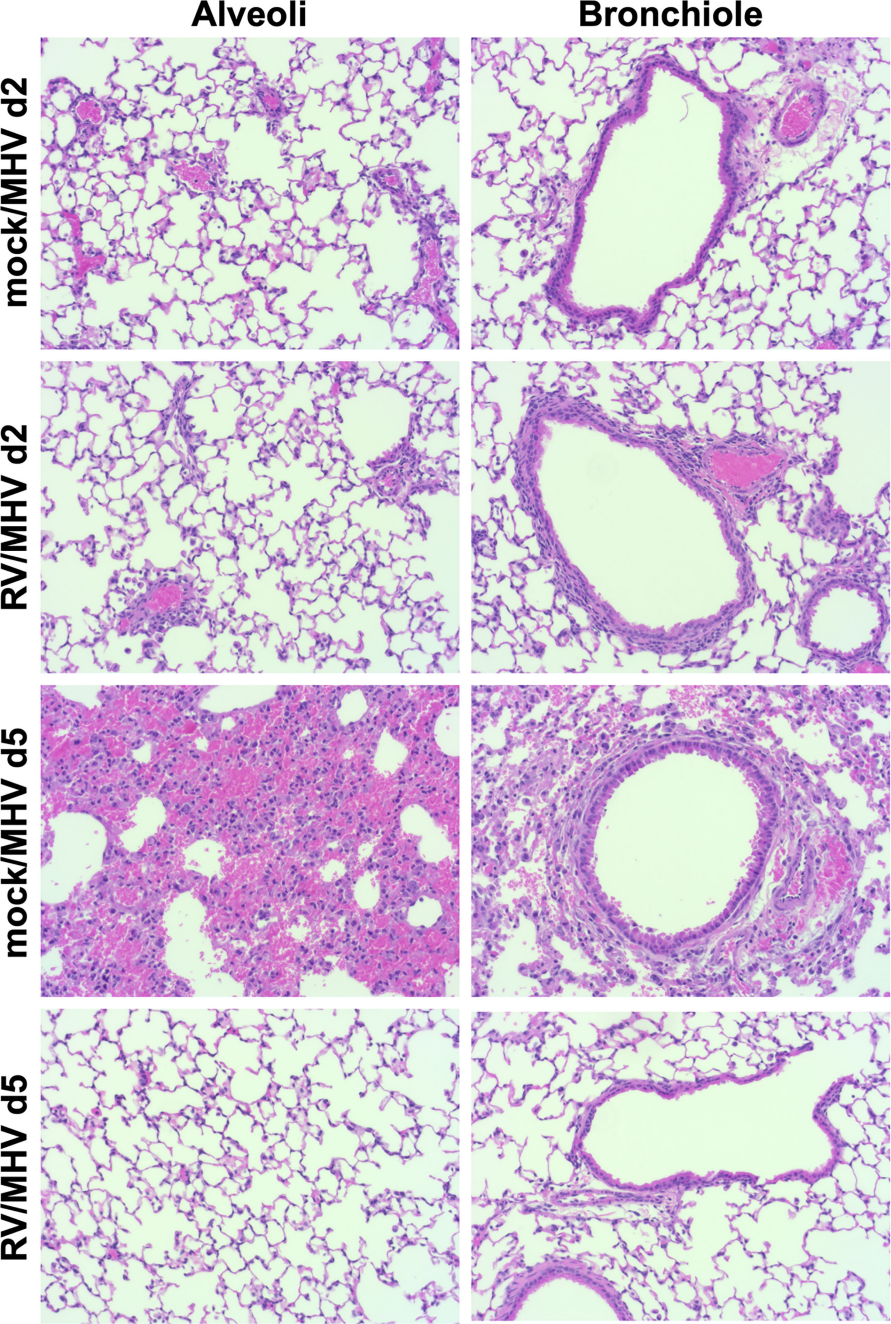
Priming with RV limits pulmonary inflammation and damage upon MHV-1 infection. Mice (n=2 per group and time point) were inoculated intranasally with RV (7.6x10^6^ TCID_50_) or saline (mock) on day -2, and MHV-1 (2x10^5^ PFU) on day 0. Lungs were collected on days 2 and 5, fixed in formalin, and 5 μm sections were stained with hematoxylin and eosin. Representative images from one mouse per group are shown at 20X magnification of alveoli and bronchioles.

We performed histopathology analysis of lung tissues to determine the effects of RV priming on inflammation and damage upon MHV-1 infection (**Figure 5**). Lung tissues from mock/MHV and RV/MHV infected mice were similar on day 2, with slightly increased peribronchiolar and perivascular cuffing in RV-primed mice. In contrast, by day 5, mock/MHV infected lungs had extensive inflammation in the alveoli and around bronchioles. Red blood cells were seen through-out the sections, indicating widespread pulmonary hemorrhage. Despite the early infiltration of immune cells on day 2 in RV-primed mice, by day 5 RV/MHV infected lung sections were clear of infiltrating leukocytes and had reduced peribronchiolar and perivascular cuffing compared to day 2. Thus, RV appeared to induce early recruitment of immune cells to the lungs and limited pulmonary inflammation and pathology induced by MHV-1 infection.

### RV Does Not Inhibit MHV-1 Replication in a Mouse Lung Epithelial Cell Line

**Figure 6:**
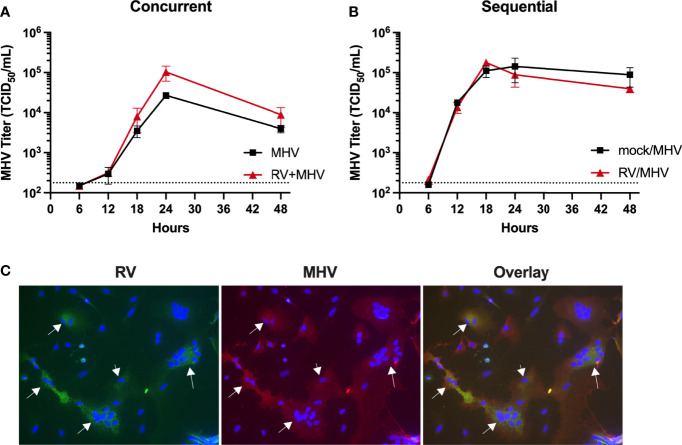
RV does not inhibit MHV-1 replication in a coinfected epithelial cell line. LA4 cells were inoculated with **(A)** RV and MHV-1 concurrently or **(B)** RV 6 hours before MHV-1. Supernatant media from triplicate samples per time point were titrated for MHV-1 by TCID_50_ assay using 17Cl.1 cells. **(C)** LA4 cells were inoculated with RV and MHV-1 concurrently, and viral antigens were labelled by IFA 18 hours later. Antibodies against RV were detected with Alexa488 (green) and MHV-1 with Alexa555 (red) and nuclei were labelled with DAPI (blue). The panels show RV (green), MHV-1 (red), and overlay of both images. White arrows show examples of coinfected cells containing both RV and MHV-1 antigens.

Other studies have shown that RV inhibits SARS-CoV-2 replication in primary respiratory epithelial cells *in vitro* (8). We tested whether RV would inhibit MHV-1 infection in a murine lung epithelial cell line, LA4. LA4 cells were inoculated with MHV-1 and RV concurrently or sequentially with RV 6 h prior to MHV-1 (**Figure 6**). In contrast to our *in vivo* findings (**Figure 2**), RV did not inhibit replication of MHV-1 either during concurrent or sequential coinfection (**Figures 6A, B**). In order to determine whether RV and MHV-1 were infecting the same cells within a coinfected culture, we performed IFA for viral antigens 18 h after concurrent coinfection. As we have previously shown, MHV-1 formed syncytia among infected cells, while cells infected with RV alone were dramatically condensed (21). Several cells contained antigens from both viruses (**Figure 6C**, arrows), indicating that neither virus inhibited super-infection of the cell by the other virus.

### RV Dominates the Transcriptional Response of Mouse Lung Epithelial Cells Over That of MHV-1

**Figure 7:**
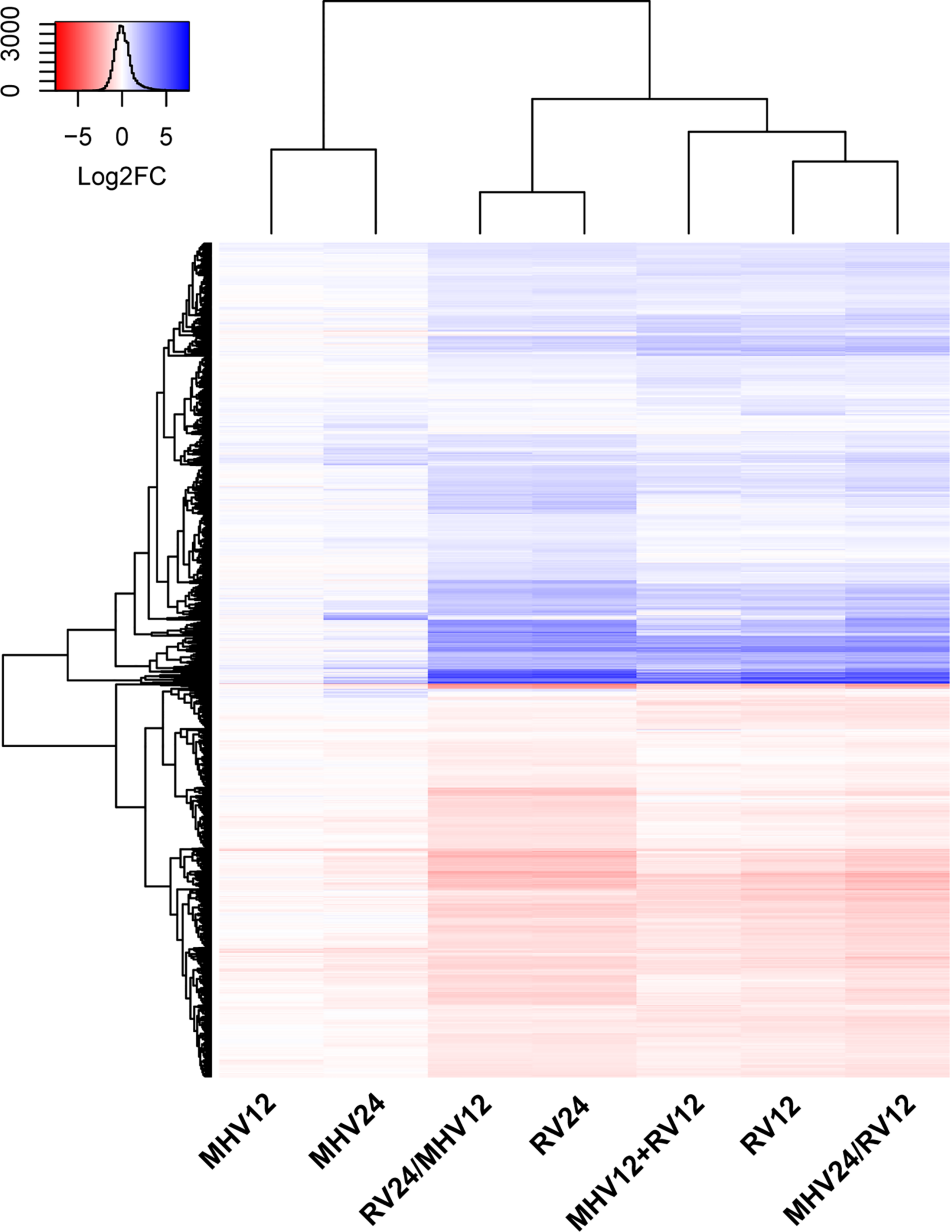
RV dominates gene expression patterns over MHV-1 in coinfected epithelial cells. LA4 cells were inoculated with RV or MHV-1 for 12 or 24 hours (RV12, RV24, MHV12, MHV24), both viruses for 12 hours (MHV12+RV12) or one virus 12 hours before the other virus (RV24/MHV12, MHV24/RV12). RNA was isolated and host gene expression was analyzed using a mouse genome microarray. Heat map includes expression of all significantly up (blue) or down (red) regulated genes compared to mock-inoculated cells with colors indicating log_2_ fold change vs. mock (see inset key). Gene and sample clustering (dendograms) were performed using hclust in R. See **Supplementary Table 1** for all significant gene expression changes for each relevant pairwise comparison.

To understand how coinfection by MHV-1 and RV affects gene expression in epithelial cells, LA4 cells were inoculated with MHV-1 alone, RV alone, or coinfected with both viruses (concurrently and sequentially) and total gene expression was analyzed using microarrays. Genes were more dramatically up- or down-regulated by RV infection at both 12 and 24 h (RV12; RV24) time points compared to MHV-1 (MHV12; MHV24; **Figure 7**). Cells coinfected with both viruses for 12 h (MHV12+RV12) had a similar gene expression profile to those infected by RV for 12 h (RV12). The difference in gene expression levels vs. mock was increased in cells infected with MHV-1 for 12 h prior to RV for an additional 12 h (MHV24/RV12) compared to RV alone for 12 h (RV12). However, when MHV-1 was added to cells 12 h after RV and gene expression was analyzed 24 h after RV inoculation (RV24/MHV12), gene expression was nearly identical to cells infected by RV alone for 24 h (RV24), seen by the similar log_2_ fold changes vs. mock (**Figure 7**) and list of differentially expressed genes (**Supplementary Table 1**). There were only 64 genes with significantly different expression in RV24/MHV12 vs. RV24, whereas 2360 genes were differentially expressed between RV24/MHV12 vs. MHV12 (**Supplementary Table 1**). Thus, RV induced stronger gene expression changes than MHV-1, as we have shown previously (21), and dominated gene expression patterns during sequential and concurrent coinfection with MHV-1.

## Discussion

While clinical and epidemiological data indicate that respiratory viruses can interfere with replication or circulation of distantly related viruses and alter pathogenesis within a coinfected host, animal models are critical for determining the immunological mechanisms that contribute to viral interference. Here, we show that inoculation of mice with RV two days before a lethal dose of MHV-1 completely protected against mortality and reduced morbidity, viral replication, inflammatory cell recruitment to the airways, and inflammation and pathology in the lungs. While RV did not inhibit replication of MHV-1 in cultured lung epithelial cells, it dominated the gene expression response of these cells to infection.

Multiple viral pairs have been found to attenuate disease upon coinfection. In addition to MHV-1, we have shown that RV protects mice against lethal infections by influenza A virus (IAV) and PVM when given two days prior to the lethal virus (14, 15). Interestingly, attenuation of IAV disease was found to be dependent on type I IFN signaling, while protection against PVM was not (15). We further showed that a non-lethal dose of MHV-1 protects mice against subsequent infection by IAV, which is associated with a robust type I IFN response induced by MHV-1 in mouse lungs (14). Similarly, others have shown that an MHV-1 infection limited to the upper respiratory tract of mice prevents mortality of a subsequent SARS-CoV infection and reduces the severity of IAV (22). They further showed that nasal priming by MHV-1 triggers type I IFN-independent recruitment of immune cells to the lungs that can then be activated upon challenge with a lethal pulmonary virus (22). A recent study found that prior infection with RSV protects mice against IAV, but not vice versa (23). In contrast to the two-day separation between viral inoculations we used, Hartwig et al. showed that protection was effective when RSV was given to mice 4, 8, or 30 days before IAV (23). This timing is much later than the type I IFN response induced by RSV, suggesting other correlates of protection are involved. Thus, different viral combinations result in attenuated disease upon coinfection, and there are multiple mechanisms responsible for disease attenuation including both type I IFN-dependent and -independent mechanisms.

Other studies in animal models have found viral pairs that exacerbate disease severity. In contrast to Hartwig et al. described above, George et al. found that IAV given to mice 24 h prior to RSV resulted in more severe disease than either virus alone (24). However, the studies differed in the severity of IAV infection and timing between viruses. In a Syrian hamster model, concurrent or sequential coinfections with SARS-CoV-2 and an H1N1 stain of IAV resulted in enhanced disease (25). In contrast, an H3N2 strain of IAV was inhibited by SARS-CoV-2 coinfection in the hamster model (26). Additional studies in hACE2-transgenic mice and ferrets observed enhanced disease upon SARS-CoV2 and influenza virus coinfection (27–31). We have shown that when RV was given two days after IAV infection, mice succumbed to the infection faster than when given IAV alone (14). In addition to virus combinations and host susceptibility, the order, timing, and doses of coinfecting viruses are likely important factors in determining if disease is enhanced or attenuated.

IFNAR signaling was required for survival upon non-lethal MHV-1 infection. However, the robust expression of IFN-β in the lungs of mock/MHV infected mice did not limit viral replication or protect from lethal disease and RV/MHV infected mice had lower levels of IFN-β. These seemingly contradictory results are likely due to the timing of the IFN response. We inhibited IFNAR signaling from the start of MHV-1 infection, thus giving the virus a head start to overwhelm other innate immune signaling. Although RV-primed mice had lower levels of IFN-β on day 2 after MHV infection (day 4 after RV priming), we previously showed that RV induces an early, albeit low, type I IFN response (14, 15). This response is likely adequate to suppress early replication of MHV-1, which results in lower IFN-β expression in contrast to mock/MHV infected mice.

The reduced leukocyte recruitment and inflammation in the lungs of RV-primed mice may be due to early suppression of MHV-1 replication, direct inhibition of inflammatory signaling, or both. We previously showed that priming with RV reduces inflammatory responses upon IAV infection, which is independent of IAV replication (14). Although we did not evaluate RV-induced cellular recruitment prior to MHV-1 inoculation, we have previously shown that RV induces low numbers of inflammatory cells into the lungs by day 2 after inoculation (15). RV-inoculated mice had similar total cells in the airways as mock-inoculated mice, but had a lower percentage of macrophages and higher neutrophil population (15). Macrophages are target cells for MHV-1 infection in mouse lungs (9), so this reduction in macrophages could limit the availability of target cells and thus MHV-1 replication. Others have shown rapid recruitment of neutrophils to the airways of RV-inoculated BALB/c mice and a return to baseline by day 4 (32). Thus, the time points we evaluated were likely after RV-induced cellular infiltration had cleared. The potential role of early recruitment and retraction of immune cells upon RV inoculation in limiting the availability of target cells for MHV-1 infection and/or excessive inflammatory responses will be evaluated in future studies. Others have shown that RV down-regulates signaling by macrophages and epithelial cells upon secondary bacterial infections, resulting in reduced neutrophil recruitment and enhanced disease (33, 34). While suppression of neutrophil responses is detrimental during bacterial infection, neutrophils can contribute to excessive pathology during respiratory viral infections, including coronaviruses (35, 36). Thus, the reduced recruitment of neutrophils in RV-primed mice may limit pulmonary damage, thereby attenuating disease severity.

Coinfection with RV did not limit replication of MHV-1 *in vitro* despite induction of a type I IFN response (**Figures 6** and **7**; **Supplementary Table 1**) (21). In contrast to our findings, others have found that RV inhibits replication of SARS-CoV-2 *in vitro* when these two viruses are inoculated simultaneously or sequentially (8, 37, 38). Furthermore, interference of SARS-CoV-2 replication was dependent on type I IFN signaling (8, 37, 38). The difference in outcomes could be due to differential sensitivity of SARS-CoV-2 vs. MHV-1 to IFN-dependent inhibition or differences in cell type specificity of inhibition. In agreement with studies using other mouse strains (12), we found that type I IFN signaling is required to protect BALB/c against a non-lethal MHV-1 infection. Additional studies have demonstrated that mouse strain-dependent differences in susceptibility to MHV-1 infection correlate with type I IFN responses (9, 12, 39). However, mechanisms besides direct inhibition of viral replication likely contribute to protection; for example, stimulation of natural killer cells, dendritic cells, or CD4^+^ or CD8^+^ T cells, or modulation of inflammatory responses (18, 40–45). Type I IFN signaling by hemopoietic cells, especially macrophages and cDCs, is critical for protection from severe disease upon infection of mice with the A59 strain of MHV (46, 47). Future studies will be important to identify potential IFN-dependent mechanisms of RV-mediated protection against MHV-1 and the cell-type specificity of these mechanisms.

We found that MHV-1 upregulated a robust type I IFN response *in vivo*, but not *in vitro* (**Figures 3** and **7**) (21). This is likely also due to cell type-specific responses. Our *in vitro* studies were done in LA4 cells, an immortalized epithelial cell line derived from murine lung tissue. This is a convenient system for our studies because it is susceptible to infection by a diverse set of respiratory viruses used in mouse model systems, including RV strain 1B and MHV-1 (21). However, MHV-1 has been reported to replicate predominantly in alveolar macrophages in mouse lungs (9). The robust type I IFN mRNAs detected *in vivo* may be expressed by MHV-1-infected alveolar macrophages and/or additional cell types responding to the infection, such as NK cells and plasmacytoid dendritic cells (12, 46). Furthermore, MHV-1 causes more severe disease in strain A/J mice, which corresponds with reduced type I IFN production (9). LA4 cells were derived from strain A/He mice (48) and thus might be expected to have reduced IFN responses to MHV-1 infection. Related strains of MHV (JHM and A59) also do not induce expression of type I IFN *in vitro* (49). However, they do not actively inhibit type I IFN production induced by other triggers, such as dsRNA (49). Like other positive-stranded RNA viruses, MHV replicates within double membrane bound vesicles in the cellular cytoplasm (50, 51). These vesicles likely hide viral dsRNA intermediates from pattern recognition receptors that would trigger expression of type I IFNs.

In summary, we observed viral interference when mice were primed with RV prior to a lethal pulmonary coronavirus infection, including complete protection from mortality. Although replication of MHV-1 was reduced in RV-primed mice, RV did not inhibit replication of MHV-1 in cultured epithelial cells, suggesting that interference involves immunological mechanisms not present in our *in vitro* system. This mouse model will be critical for identifying cellular and molecular mechanisms of viral interference that may explain observations of altered disease severity in coinfected patients.

## Data Availability Statement

The datasets presented in this study can be found in online repositories. The names of the repository/repositories and accession number(s) can be found below: https://www.ncbi.nlm.nih.gov/geo/, Accession numbers: GSE89190 and GSE201471.

## Ethics Statement

The animal study was reviewed and approved by the Institutional Animal Care and Use Committee, University of Idaho.

## Author Contributions

TM and CM contributed to conceptualization, study design, and procured funding. GC, AG, EI, AR, and TM performed experiments, assays, and data analysis. JVL and TM performed statistical analyses and generated figures. TM wrote first draft of the manuscript. All authors contribute to manuscript revision and read and approved the submitted version.

## Funding

This work was supported by the National Institute of General Medical Sciences of the National Institutes of Health awards P20 GM104420 and P20 GM103397.

## Conflict of Interest

The authors declare that the research was conducted in the absence of any commercial or financial relationships that could be construed as a potential conflict of interest.

## Publisher’s Note

All claims expressed in this article are solely those of the authors and do not necessarily represent those of their affiliated organizations, or those of the publisher, the editors and the reviewers. Any product that may be evaluated in this article, or claim that may be made by its manufacturer, is not guaranteed or endorsed by the publisher.

## Supplementary Material

Supplementary Table 1 Microarray data presenting all significantly different genes in each pairwise comparison.
